# Genomic Characterization of Bacteriophage Babydotz, Isolated from *Microbacterium foliorum*

**DOI:** 10.17912/micropub.biology.001869

**Published:** 2026-08-10

**Authors:** Harris McFerrin, Dahlia Ahmed, Justice Austin, Classie Baker, Skyler Brown, Kaionah Cooper, Kennedy Drake, Liyah Galbert, Jillian Harris, Nora Herrera, Gerald Jones, Camerin Kimble, Alicia Lewis, Leianna Yancey, Michael Khalil, Shaundessy Perry

**Affiliations:** 1 Department of Biology, Xavier University of Louisiana, New Orleans, Louisiana, United States

## Abstract

Microbacteriophage Babydotz was isolated in Moorhead, Minnesota in 2020 by infecting *Microbacterium foliorum* NRRL B-24224. Based on gene content, is is assigned to actinobacteriophage cluster EG. With no identifiable genes involved in lysogeny, it is predicted to have a lytic life cycle.

**Figure 1. Transmission electron microscopy (TEM) of microbacteriophage Babydotz:** TEM of uranyl acetate-stained microbacteriophage Babydotz shows a siphovirus morphology.

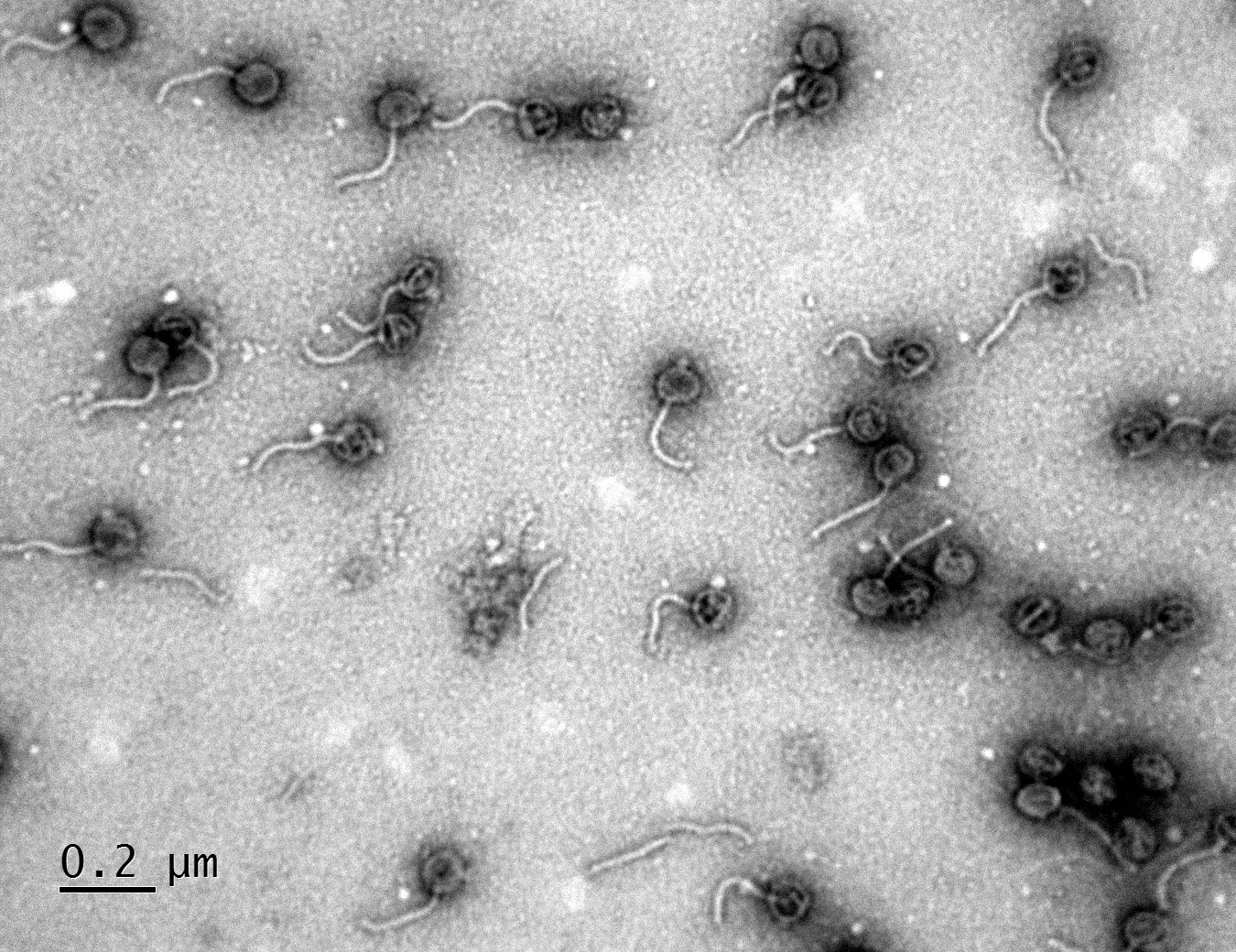



## Description

Research on bacteriophages has contributed to our understanding of viruses more broadly and in their development as therapeutics for multi-drug resistant bacterial infections (Kim et al, 2025). Here, we report the genome sequence of Babydotz, a microbacteriophage that infects *Microbacterium foliorium.*

Babydotz was isolated from a soil sample that was collected between two pine trees at the Minnesota State University Moorhead Campus Mall (global positioning system [GPS] 46.8673 N, 96.762 W) when the ambient temperature was -2°C. Briefly, Babydotz was extracted by washing the soil with PYCa liquid medium, filtering the wash, and plating the filtrate in top agar with *M. foliorum* NRRL B-24224 (Zorowik et al, 2024). After incubation at 30˚C for 24 hours, Babydotz formed clear plaques with a diameter of 0.67-0.75mm (n=5), measured using ImageJ software (Schneider, et al, 2012). Negative-staining transmission electron microscopy showed siphovirus morphology with a capsid diameter of 80-90nm (n=5) and tail length of 180-200nm (n=5) (Figure 1).

DNA was extracted from a Babydotz lysate using a Promega Wizard DNA extraction kit prior to sequencing. The Pittsburgh Bacteriophage Institute generated sequencing libraries using the NEB Ultra II FS Kits and sequenced the genome on an Illumina MiSeq using 150-cycle v3 Reagent Cartridges (Russell DA, 2018). Sequencing yielded 613,595 150-base single-end reads. A single bacteriophage contig with 1387-fold coverage was assembled from the raw reads using Newbler v.2.9 and Consed v29 with default parameters (Russell DA, 2018). The resulting genome consisted of 63,076 bp, 217-bp direct terminal repeats and 67.0% G+C nucleotides.

Auto-annotation was performed using DNA Master v5.23.6 (Pope et al., 2018) which employs GeneMark v.3.02 (Delcher et al., 1990) and Glimmer v.2.5p (Borodovsky et al., 2003; Besemer et al., 2005). DNA Master, Phamerator, using Actino_draft database v578 (Cresawn et al., 2011) and PECAAN (Rinehart et al., 2016) were used to manually refine the annotations. Starterator (Pacey, 2014) was used to further evaluate start sites conservation with Glimmer and Genemark. When there was disagreement among these software programs, BLAST results, coding potential and gene length were also used to determine the most likely start for the gene. No tRNA coding genes were identified using tRNAscan-SE v2.0 (Lowe, 2016) or ARAGORN v1.2.41 (Laslett, 2004). Putative functions for 23 of 104 predicted coding ORFs were assigned using BLASTp with an e-value cutoff of 10 ^-4^ , using the Actinobacteriophage and NCBI non-redundant database (Altschul et al., 1990) and HHPRED, using the PDB_mmCIF70, Pfam- v.36, NCBI Conserved Domains databases with a probability of 90% or greater (Zimmermann et al., 2018; Söding et al., 2005). Nine protein-coding ORFs with membrane-associated domains were determined using SOSUI (Hirokawa et al., 1998) and TMHMM v.1.0.24 (Hallgren et al., 2022). All tools were run with default parameters. Following student genome annotation, the annotation underwent peer review by an experienced SEAPhage-associated faculty and passed quality control inspection. AUG start codon usage was 100%. BabyDotz was assigned to cluster EG based on gene content similarity of at least 35% to phages in the Actinobacteriophage database, phagesdb ( https://phageDB.org ) (Pope et al., 2017; Russell and Hatfull, 2017). Phamerator was used to compare genome architecture and gene synteny between Babydotz and other cluster EG phages. Except for several genes near the left end of the genome, Babydotz exhibits strong synteny with other peer-reviewed cluster EG phages with which there was high sequence alignment (BLASTn E value, Score) including Rowlf (0.0, .93e+04), SallieK (0.0, 1.697e+04), Tissue (0.0, 1.687e+04) and Zagie (0.0, 1.657e+04), consistent with its assignment to cluster EG. Consistent with other cluster EG phages, the central third of the Babydotz genome is transcribed in the forward direction, whereas the flanking regions are transcribed in the reverse direction. An endolysin is encoded immediately upstream of the boundary where transcription switches from the reverse to the forward direction, consistent with the genomic organization observed in other cluster EG phages including Rowlf, SallieK, Tissue and Zagie.

Other predicted genes include those encoding a putative DprA-like DNA processing chain A, three helix-turn-helix DNA binding domains, a terminase, a portal protein, a major capsid protein, a major tail protein, a phosphoesterase, a head-to-tail adaptor, a tape measure protein, three minor tail proteins, an endolysin, an exonuclease, an HNH endonuclease, a RuvC-like resolvase, a MazG-like nucleotide pyrophosphohydrolase, an SSB protein, a DNA primase/helicase and two ribbon-helix-helix DNA binding proteins. No genes encoding immunity repressor or integrase functions could be identified, suggesting that Babydotz is unlikely to establish lysogeny.

**Nucleotide sequence accession numbers**

Babydotz is available at GenBank with Accession No. 0R613478 and Sequence Read Archive (SRA) No. SRR18306109.
